# Preparation of Antimony-Doped Tin Oxide Fly Ash Antistatic Composite and Its Properties in Filling EVA

**DOI:** 10.3390/ma17051183

**Published:** 2024-03-03

**Authors:** Ying Qiu, Caili Wang, Chunxue Zhao, Guoxin Yao, Zhixue Wang, Runquan Yang

**Affiliations:** College of Mining Engineering, Taiyuan University of Technology, Taiyuan 030024, China; qy1598665204@163.com (Y.Q.); 15681158727@163.com (C.Z.); massfree@163.com (G.Y.); 15340692638@163.com (Z.W.); yangrunquan@tyut.edu.cn (R.Y.)

**Keywords:** fly ash, antimony-doped tin oxide, antistatic properties, EVA, mechanical properties

## Abstract

As a common coal-based solid waste, fly ash is widely used in material filling. However, due to the high resistivity of fly ash itself, the antistatic performance of the filling material is poor. Therefore, antistatic composite powder was prepared by coating nano-sized antimony-doped tin oxide (ATO) on the surface of fly ash, and its preparation mechanism was discussed. The composite powders were characterized by SEM, EDS, XRD and FTIR. The results show that the interaction between SiO_2_ and SnO_2_ appears at the wave number of 727.12 cm^−1^, and the obvious SnO_2_ crystal phase appears on the surface of fly ash. The volume resistivity of calcined fly ash is 1.72 × 10^12^ Ω·cm, and the volume resistivity of ATO fly ash is reduced to 6 × 10^3^ Ω·cm. By analyzing the limiting oxygen index, melt index, tensile strength, elongation at break, cross-section morphology and surface electrical resistivity of EVA, it was found that the addition of antistatic powder to EVA can improve its antistatic performance without deteriorating the mechanical properties of EVA.

## 1. Introduction

Ethylene-Vinyl Acetate (EVA), whose chemical formula is (C_2_H_4_)x(C_4_H_6_O_2_)y, belongs to polyolefin compounds. EVA is characterized by its commendable flexibility, resistance to chemical corrosion, and favorable weatherability. These attributes led to its widespread application in household appliances, electrical wires and cables [1]. As an insulating material, the better the insulation performance of EVA, the weaker the electrical conductivity and the greater the electrical resistivity. During usage, friction and inductive charging often result in the accumulation of electrical charges. In cases where the material has a low capacitance, even a small aggregation of charges can generate significant electrostatic voltage. When the accumulated voltage is higher than 500 V, it will cause a spark discharge. EVA polymer material is a metal-skeleton organic matter composed of a carbon skeleton. The limiting oxygen index (LOI) is only 17~19% (materials with a limiting oxygen index of less than 22% are generally considered to be flammable). In the presence of a heat source, EVA is highly susceptible to ignition. The combustion process of EVA results in substantial heat release, the formation of molten drips, and the generation of voluminous black smoke replete with noxious odors. EVA, like most polymers, is not antistatic and exhibits poor flame retardancy, which limits its application in fields such as domestic appliances, the construction industry, decorative materials and electrical wiring and cables. Therefore, it is particularly important to study the antistatic properties of EVA.

There are two main methods to reduce the electrical resistivity of materials: adding an antistatic agent and external coating. Yan et al. added functional material PPMPPE to an EVA matrix to explore the effect of additives on the flame retardancy and mechanical properties of EVA [2]. The results show that the flame retardancy of EVA had been greatly improved, and its mechanical properties were well maintained. So, it is feasible to add an antistatic agent to EVA to improve the resistivity of the material. Metal oxides are relatively common antistatic agents, mainly tin oxide, indium oxide and zinc oxide [3,4]. At room temperature, these metal oxides belong to a range of insulating materials. Studies have shown that the introduction of impurity atoms in metal oxides generates carriers [5,6], which will improve the conductivity. For example, impurities such as antimony, phosphorus, fluorine and chlorine are added to tin oxide. Among them, nano antimony-doped tin oxide (ATO) has been widely used because of its excellent antistatic properties [7,8].

Fly ash is a solid ash produced by coal used in power generation, heating, metal smelting and other processes [9]. The main chemical components of fly ash are SiO_2_ and Al_2_O_3_, containing a small amount of CaO, Fe_2_O_3_, K_2_O, MgO and so on. The fly ash contains hollow microspheres, which exhibit a high refractive index and large reflection coefficient, thus reducing thermal radiation and convection [10]. The volume resistivity of fly ash is approximately 1.72 × 10^12^ Ω·cm, which is stronger than that of insulating materials. The annual increase in fly ash production, if not judiciously utilized, poses substantial risks to human activity and the environment. Therefore, the promotion of its application in large-volume and high-value-added products is imperative to enhance resource utilization efficiency. In an effort to maximize resource usage while simultaneously improving the flame retardancy and antistatic properties of EVA, fly ash has been employed as a filler to boost its antistatic characteristics. Li et al. [11] used small-particle-size microspheres as fillers to fill epoxy resin, and found that the flexural strength of the filled epoxy resin was improved, and as the filling amount increased, the corresponding flexural strength increased. Porabka et al. [12] filled fly ash hollow microspheres into polymers. The results show that fly ash can improve the flame retardancy of polymers to a certain extent. Yang et al. [13] doped different contents of an Al element into zinc oxide powder to prepare a nano-AZO powder and filled it into polyethylene terephthalate (PET) to improve the antistatic properties of PET. The results showed that when the filling amount of AZO was 0.005%, the surface electrical resistivity of the composite was the lowest, which was 1.16 × 10^10^ Ω/m^2^. Therefore, it can be seen from previous studies that fly ash can improve the mechanical, thermal and antistatic properties of the matrix when it is used as a filler to fill the polymer. However, the antistatic performance of unmodified fly ash-filled polymer does not meet the application requirements.

Due to the poor dispersibility of nano antimony-doped tin oxide [14], agglomeration will occur when it is directly filled in EVA, which will reduce the mechanical properties of EVA. Therefore, many scholars have coated nano-ATO on well-dispersed powders to improve the dispersibility of nano antimony-doped tin oxide and reduce the cost of antistatic agents. Zhang et al. [15] successfully coated ATO on the surface of SiO_2_ powder by a chemical-precipitation method. Wang et al. [16] used the chemical-precipitation method to coat ATO on the surface of wollastonite and successfully prepared antistatic composite powders with good dispersion and low resistivity.

Therefore, in this paper, fly ash with excellent physical and chemical properties is used as the matrix [17], and the fly ash surface is coated with nano-ATO to prepare an antistatic powder for filling EVA. By testing the mechanical properties and antistatic properties of composite materials, the performance enhancement of fly ash-based antistatic materials on EVA was explored. It is hoped that these works can bring some enlightenment to the optimization of the preparation of fly ash-based antistatic powder, so as to prepare modified fly ash with a better performance and promote the application of fly ash in antistatic fillers.

## 2. Materials and Methods

### 2.1. Materials

Fly ash was provided by Shanghai Green Sub-nanoseale Material Co., Ltd. in Shanghai, China, with a particle size of 3.58 microns and a fineness of 1250 mesh. The fly ash used in this paper is low-calcium fly ash, and the CaO content is 3.5%. It belongs to F-grade for fly ash and has good durability and fluidity when filled with polymer. Table 1 shows the chemical composition of fly ash. The main chemical elements of fly ash are Si and Al, and their mass fractions are 50.00% and 23.67%, respectively. SnCl_4_·5H_2_O, SbCl_3_ and NaOH were purchased from Aladdin limited Company (Cuddington, UK). EVA (brand 670, VA content 12%) was purchased from Dow DuPont (Wilmington, DE, USA).

### 2.2. Preparation of Composite Powder

**Figure 1:**
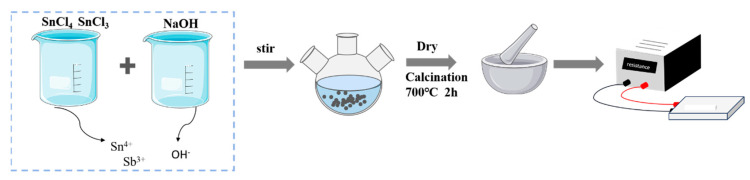
Laboratory equipment drawing.

The nano-ATO was coated on the calcined fly ash by chemical-precipitation method. After calcination, the fly ash-based antistatic composite powder was obtained. The specific test conditions and equipment are shown in Figure 1. The fly ash was calcined at 700 °C to remove the unburned carbon particles on the surface of the fly ash. Subsequently, the calcined fly ash was prepared into a solution and placed in a three-necked flask. The ratio of fly ash to water was 1:4. The molar ratio of SbCl_3_ and SnCl_4_·5H_2_O mixed solution is 1:6, and the coating amount is 25%.

In the preparation of mixed solution, a certain amount of acidic substances should be used to inhibit the precipitation of mixed solution and water. From the previous experiments, it was found that the hydrochloric acid solution with the concentration of 1 mol/L and 5 mol/L could not inhibit the precipitation. When the concentration of hydrochloric acid increased to 11 mol/L, the precipitate disappeared. Therefore, the hydrochloric acid solution with a mass fraction of 36–38% was used. The mixed solution and NaOH solution were added with a constant current pump at a dropping speed of 1 mL/min, and heated in a water bath at 60 °C. After the solution was added, the pH value of the solution was adjusted to 5, and the reaction was continued for 30 min. After cooling and settling, the stirrer was closed, washed with a large amount of water and dried, and then calcined at 700 °C for 2 h in a muffle furnace to obtain antistatic powder. The electrical resistivity of the composite powder was measured by the resistance measuring meter shown in Figure 1.

### 2.3. Preparation of EVA Composite Material

The calcined fly ash and ATO@fly ash were blended with EVA in a twin-screw extruder at a mass fraction of 30%. The screw speed was 300 r/min and the feeding speed was 30 r/min. The extruder had 10 heating sections from the feed port to the discharge port, and the temperatures are 110, 115, 120, 125, 130, 135, 140, 145, 150 and 155 °C. Single-screw injection-molding agent was used for injection molding. The injection temperature was 110~150 °C and the injection pressure was 50~60 MPa. The prepared material was placed at room temperature for 24 h. The tensile strength, elongation at break, limiting oxygen index, melt index and resistance of the material were tested. Each group was tested with a total of 5 samples, and the average value of the measurement was taken.

### 2.4. Testing Methods

In this experiment, the fly ash samples were sprayed with gold by using the British Quorum SC7620 sputtering coating instrument. The spraying time was 45 s and the thickness was 10 nm. German ZEISS Sigma 300 scanning electron microscope was used to measure the microstructure of the powder before and after modification. German ZEISS Sigma 300 was used to test the surface element distribution map and semi-quantitative sample composition data of antistatic powder. The sample was coated on a metal tray with conductive adhesive tape. After gold spraying, the sample was measured by FEI Talos F200X G2 transmission electron microscope. The crystal phase of the composite powder was measured by Rigaku MiniFLex600 X diffractometer. The target was copper and the wavelength was 5~80°. The chemical bonds or functional groups in the sample molecules were measured using German Bruker Tensor27 Fourier transform spectrometer with a test resolution of 4 cm^−1^. The KBr and the powder were dried and ground together to less than 2 μm for tableting and then placed in the instrument for measurement. The electrical resistance of the composite powder was measured by the GEST-121A resistance-measuring instrument of China Beijing Guance Co., Ltd. (Beijing, China). The electrode area A is 10 cm^2^ and the powder thickness h is 1 cm. The calculation formula of volume electrical resistivity is shown in Equation (1).

(1) $$\rho_{V}=R_{V}\frac{A}{h}$$

where *ρ_V_* is the volume electrical resistivity, Ω; *R_V_* is volume electrical resistance, Ω; *A* is the area of the electrode, cm^2^; and *h* is the thickness of powder, cm.

The tensile strength and elongation at break of the material were tested by CMT6104 universal testing machine of Wanchen Testing Machine Co., Ltd. (Jinan, China). The melt index of the material was measured using CZ-6001B melt flow-rate meter of Changzhe test Machinery Co., Ltd. (Yangzhou, China). The surface resistance R_s_ of the material was measured directly using SM7110 high-resistance meter of Rizhi (Shanghai) Measurement Technology Co., Ltd. (Shanghai, China). The electrical resistance test range is 1 × 10^3^ Ω~2 × 10^19^ Ω. The surface electrical resistivity of the material can be calculated by Equation (1). The tensile fracture morphology of the material was observed by German Zeiss Sigma 300. The limiting oxygen index of the material to maintain combustion was tested using JF-3A digital oxygen-index tester of Zhonghang times instrument equipment Co., Ltd. (Beijing, China).

(2) $$\rho_{S}=R_{s}\frac{d}{g}$$

where *ρ_S_* is the surface electrical resistivity, Ω; *R_S_* is surface electrical resistance, Ω; *d* is the perimeter of the electrode, cm; and *g* is the distance between electrodes, cm.

## 3. Results and Discussion

The electrical resistance *R_V_* of different powders was measured using a GEST-121 A resistance-measuring instrument. The electrical resistance of calcined fly ash and ATO fly ash was 1.72 × 10^11^ Ω and 6 × 10^2^ Ω, respectively. The volume resistivity of the composite powder can be calculated using formula (1). The surface electrical resistivity of pure EVA, calcined fly ash/EVA and ATO fly ash/EVA were directly measured using SM7110 high-resistance meter, and were 6.0 × 10^14^ Ω, 1.95 × 10^14^ Ω and 4.0 × 10^8^ Ω, respectively. The formula (2) can be used to calculate the surface resistivity of different kinds of EVA.

**Figure 2:**
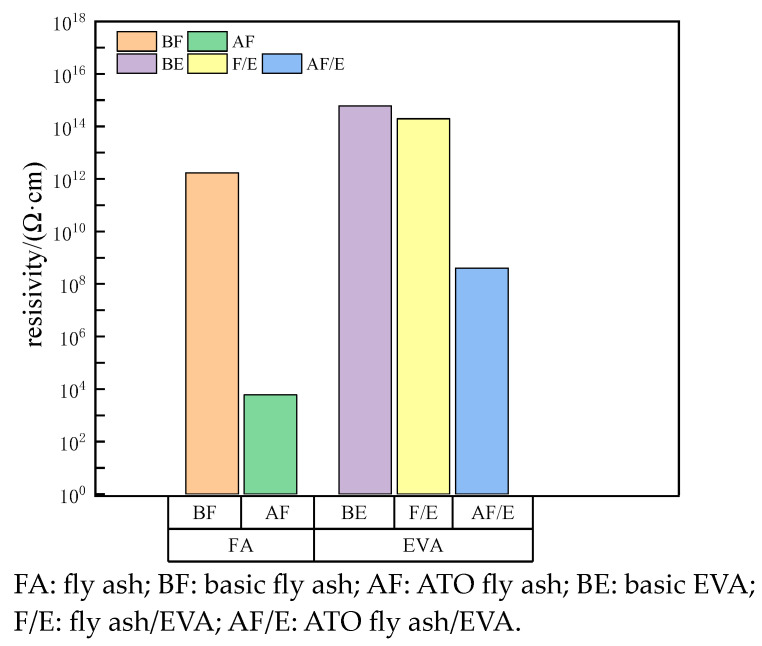
Powder, EVA electrical resistivity histogram.

As can be seen from Figure 2, the volume electrical resistivity of calcined fly ash (BF) is 1.72 × 10^12^ Ω·cm, and the electrical resistivity is high, so its antistatic performance is poor. The volume electrical resistivity of fly ash (AF) after surface coating is 6 × 10^3^ Ω·cm, which belongs to antistatic powder. After filling EVA with calcined fly ash and ATO fly ash, the volume electrical resistivity of EVA decreased to varying degrees. However, it can be seen from Figure 2 that the surface electrical resistivity of EVA filled with modified fly ash decreases greatly compared with EVA filled with calcined fly ash, and the surface electrical resistivity of modified EVA decreases to 4.0 × 10^8^ Ω. Therefore, EVA coated with modified fly ash can obtain better antistatic properties.

### 3.1. SEM and EDS Analysis of Composite Powder

**Figure 3:**
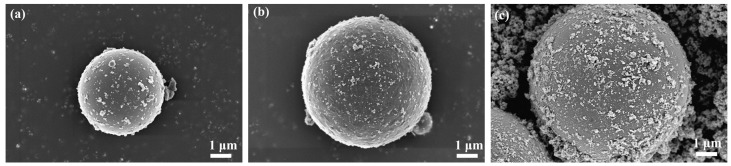
SEM image of fly ash (**a**), fly ash calcined at 700 °C (**b**), antistatic composite powder (**c**).

Figure 3a presents the fly ash produced after coal combustion. It can be seen from the scanning electron microscope (SEM) image that the fly ash is a spherical particle, and the surface contains unburned carbon particles. Figure 3b shows the SEM image of fly ash post-calcination at 700 °C, where the emergence of needle-like mullite on the surface of the fly ash is evident. Figure 3c shows the SEM image of calcined fly ash coated with nano antimony-doped tin oxide, and the ATO fly ash composite power remains spherical shape. ATO is uniformly coated on the surface of fly ash, with few exposed surfaces, and the surface roughness of fly ash increases. Therefore, the volume resistivity of the antistatic composite powder is reduced, reaching the standard of the antistatic powder [18].

**Figure 4:**
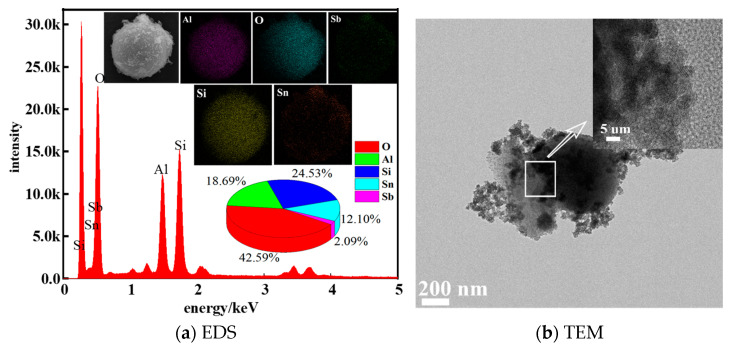
EDS diagram and TEM diagram of composite powder.

Figure 4a is the element distribution of antimony-doped tin oxide on the surface of fly ash when the coating amount is 25%. As can be seen from Table 1, fly ash contains Al, Si and O elements, but does not contain Sb and Sn elements. However, it can be seen from Figure 4 that Sn and Sb elements appeared on the surface of the composite powder after surface coating, accounting for 2.09% and 12.11%, respectively. The proportion of the two elements is similar to the molar ratio of Sb–Sn when coated with fly ash. Therefore, when the chemical precipitation method is used to coat the fly ash, the loss of the coating agent is less. The elemental scanning image taken by EDS shows that Sb and Sn are evenly distributed on the surface of fly ash. Figure 4b is the TEM image of ATO fly ash. The spherical fly ash appears dark, contrasting with the lighter outer ATO layer, indicating complete coverage. The lattice spacing (d) measured by Image J is 0.29 nm, which is consistent with the lattice spacing of SnO_2_ [19]. It can be seen from Figure 4a,b that the surface of fly ash is successfully coated with ATO.

### 3.2. XRD Analysis of Composite Powder

**Figure 5:**
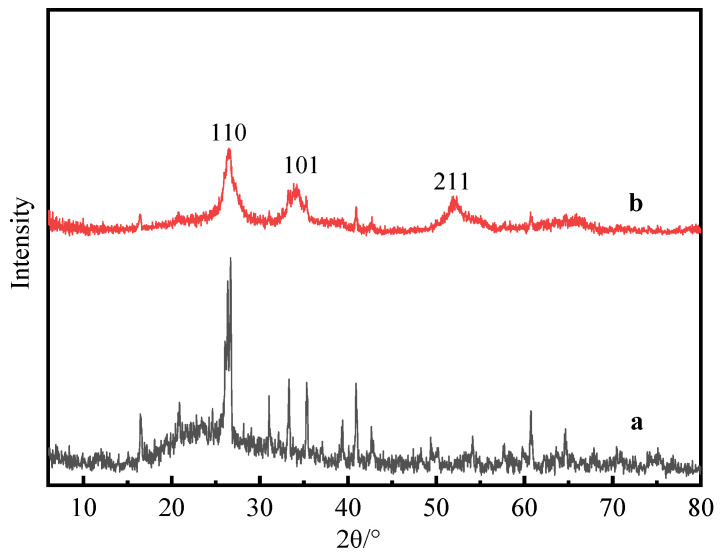
XRD pattern of fly ash (**a**) and composite powder (**b**).

Figure 5 is the XRD pattern of calcined fly ash and composite powder. Figure 5a shows the XRD spectrum of calcined fly ash, with its primary crystalline phases including mullite, sillimanite, and quartz. Figure 5b is the XRD pattern of calcined fly ash surface-coated with nano antimony-doped tin oxide (ATO). Compared with calcined fly ash, the intensity and position of the ATO diffraction peak changed significantly. As can be seen from the figure, there are obvious SnO_2_ (JCPDS: 41-1445) crystal phases on the (110), (101) and (211) crystal planes, and similar results were obtained also by other authors [20]. However, due to the low doping amount of antimony, there is no obvious antimony crystal phase.

### 3.3. FTIR Analysis of Composite Powder

**Figure 6:**
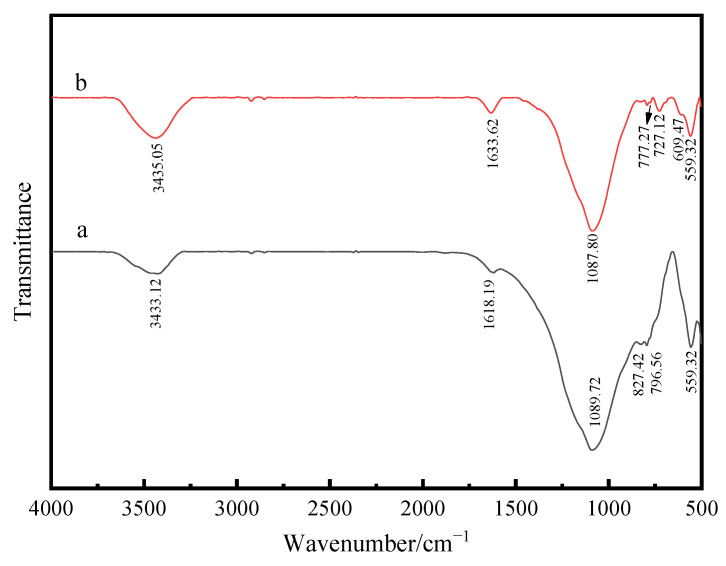
FTIR diagram of fly ash (**a**) and composite powder (**b**).

Figure 6 is the infrared absorption spectra of calcined fly ash and composite powder. Figure 6a, representing the infrared spectrum of calcined fly ash, identifies the stretching vibration and bending vibration of O-H at wavenumbers of 3433.12 cm^−1^ and 1618.19 cm^−1^ [21]. The bending vibration of Si-O appears at the wave number of 559.32 cm^−1^ and the stretching vibration of Si-O-Si appears at the wave number 1089.72 cm^−1^ [22,23]. Figure 6b is the infrared absorption spectrum of ATO fly ash composite powder. The O-H-stretching vibration appears at the wave number of 3435.05 cm^−1^. Compared with Figure 6a, the O-H peak of the composite powder moves, and the stretching vibration intensity of the O-H of the composite powder increases. This is because the chemical precipitation method is carried out in an aqueous solution, and the water contains ionized H^+^ and OH^−^, so the O-H-stretching vibration intensity of the composite powder increases accordingly. The Sn-O-Sn antisymmetric peak of SnO_2_ appears at 609.47 cm^−1^, and a new peak emerges at 727.12 cm^−1^, which is the interaction between amorphous SiO_2_ and SnO_2_ [24,25]. The Si-O-Si-stretching vibration absorption peak appears at the wave number of 1087.80 cm^−1^. Compared with the FTIR of calcined fly ash in Figure 6a, the wave number of Si-O-Si decreases from 1089.72 cm^−1^ to 1087.80 cm^−1^, which is due to the combination of SiO_2_ with Sn^4+^ and Sb^3+^ to generate Si-O-Sn (Sb).

### 3.4. Preparation Mechanism of Composite Powder

**Figure 7:**
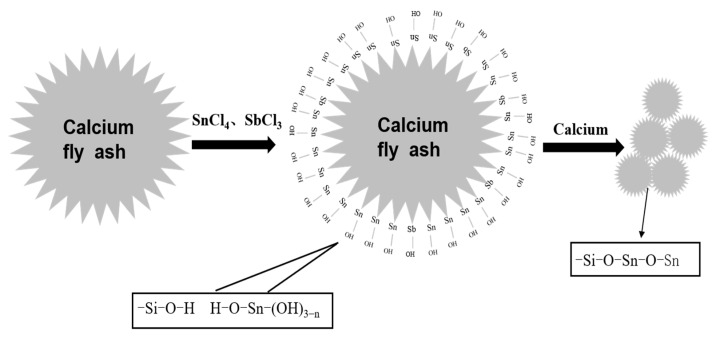
Mechanism diagram of ATO fly-ash composite powder.

The mixed solutions of SnCl_4_ and SbCl_3_ and NaOH were added dropwise into a fly-ash suspension. Sb^3+^ and Sn^4+^ reacted with the water in the fly-ash suspension to form SbO^+^ and Sn(OH${)}_{4-n}^{n+}$, which were deposited on the surface of the fly ash [26]. The reaction equations of this process are (3) and (5). After washing, precipitation and filtration, it was transformed into Sb_2_O_3_ and SnO_2_·2H_2_O. The reaction equations are shown in (4) and (6). The Si-O-Si bond in the fly-ash solution was ionized to generate (Si-O)^−^ and Si^+^, and the ionized H^+^ and OH^−^ in the water combined with it to generate Si-OH. The O-H bond is also partially ionized and converted into (Si-O)^−^, which makes the fly ash negatively charged and attracts the positively charged Sn^4+^ and Sb^3+^ in the solution, so the Si-O-Si-stretching vibration intensity decreases in FTIR. In addition, the remaining Si-OH will condense with the surface group Sn(Sb)^−^OH of the Sb-Sn hydrolysate to form the Si-O-Sn (Sb) bond [27]. Sb-SnO_2_ with a rutile structure is formed after high-temperature calcination, which has a conductive function [28]. The specific reaction Equations (3)–(6) and mechanism of the formation process of antimony-doped tin oxide in the antistatic layer on the surface of the fly ash are shown in Figure 7.

Sb^3+^ + H_2_O = SbO^+^ + 2H^+^ (3)

Sb_4_O_5_Cl_2_ + H_2_O = 2Sb_2_O_3_ + 2HCl (4)

(5) $${\mathrm{Sn}}^{4+}+\left(4-n\right)H_{2}O+\left(n-4\right)H^{+}=\mathrm{Sn}{\left(\mathrm{OH}\right)}_{4-n}^{n+}\;n=1–4$$

Sn^4+^ + 4H_2_O = SnO_2_·2H_2_O + 4H^+^ (6)

### 3.5. Performance Analysis of EVA Filled with Composite Powder

Table 2 shows the test results for tensile strength, elongation at break, melt flow index, surface resistivity, and limiting oxygen index (LOI) of pure EVA, calcined fly ash/EVA, and ATO fly ash/EVA samples. The tensile strength of calcined fly ash and ATO fly ash-filled EVA composites is enhanced compared with pure EVA. This is because the fly ash itself has the characteristics of high strength, and the specific surface area of the coated fly ash increases, which strengthens the interaction between the fly ash and the EVA [29]. The maximum tensile force that the material can withstand increases, so the tensile strength increases.

The elongation at break refers to the ratio of the elongation length of the material at the time of breaking to the original length before stretching, which can reflect the toughness and ductility of the material [30]. When EVA was filled with the two powders, the elongation at break both decreased. The surface of the calcined fly ash is not modified, so the dispersion of the material is poor. This phenomenon causes local stress concentration, which will lead to the local failure of the material and a greater reduction in elongation at break [31]. After the surface of the fly ash is coated with ATO, the surface roughness increases; thus, the elongation at the break is improved compared with the modified fly ash.

The melt index refers to the weight of the standard capillary through which the melt passes within 10 min under certain conditions. The higher the melt index, the better the processability and fluidity [32]. The melt index of EVA filled with calcined fly ash is obviously improved. This is because the calcined fly ash is spherical and the surface is smooth, which improves the fluidity of EVA and the (melt) index [33]. The melting index of EVA filled with ATO fly ash was significantly lower than that of pure EVA. This is because the surface of fly ash is coated with ATO, which makes the surface of the powder become rough, enhancing the adhesive force between the powder and EVA. Therefore, the fluidity and the melt index of EVA are reduced.

The surface electrical resistivity of pure EVA is 6.0 × 10^14^ Ω, which only changes slightly to 1.95 × 10^14^ Ω after filling with calcined fly ash. The surface electrical resistivity of EVA filled with ATO fly ash is significantly reduced to 4.0 × 10^8^ Ω, indicating a substantial improvement in EVA’s antistatic properties.

The limiting oxygen index is the oxygen concentration required for the material to reach the ignition point [34]. When the limiting oxygen index is less than 21%, it is flammable material. When the limiting oxygen is between 21% and 27%, it belongs to slow-burning material. The limiting oxygen index of pure EVA is 19.8%, categorizing it as a flammable material, and it was increased to 22% after being filled with calcined fly ash. This is because the main chemical elements of fly ash are Si and Al, with mass fractions of 50.00% and 23.67%, respectively. During the combustion process, Si(OH)_4_ and Al(OH)_3_ are generated, which can promote the charring process. The formed carbon layer can effectively block heat and smoke. The hydroxide absorbs heat when it is heated [35,36], which reduces the temperature of the system, so the limiting oxygen index was increased [37]. After filling with ATO fly ash, the LOI of EVA was further increased to 23.5%. This increase is due to Sb_2_O_3_ reaching its melting point at 665 °C and forming a protective film on the matrix surface, isolating it from the air. Sb_2_O_3_ will undergo an endothermic reaction, which will reduce the combustion temperature. Therefore, fly ash and ATO can achieve a synergistic flame-retardant effect [38,39,40].

### 3.6. Analysis of Cross-Section Morphology of EVA Filled with Composite Powder

**Figure 8:**
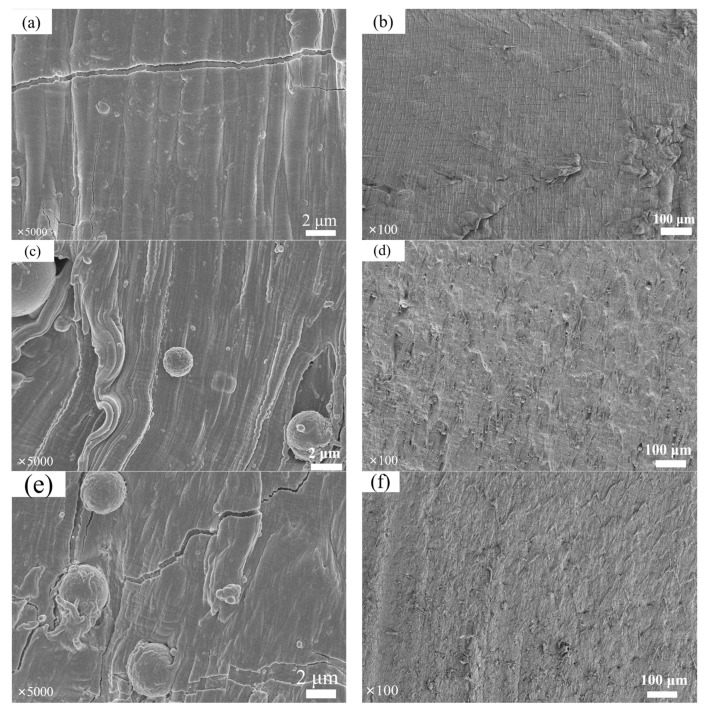
Tensile profile of pure EVA and EVA filled with different fillers. Pure EVA (**a**,**b**); calcined fly ash/EVA (**c**,**d**); ATO fly ash/EVA (**e**,**f**).

As can be seen from Figure 8a,b, the longitudinal gully of pure EVA is obvious, and the phenomenon of cross-section wire drawing is more serious, indicating that the interaction force between matrix molecules is strong [41,42]. The fracture surface morphologies of EVA filled with calcined fly ash and ATO fly ash, as shown in Figure 8c–f, demonstrate that the surface wire drawing phenomenon is enhanced and the tensile strength was increased after filling the EVA. A large number of spherical particles appeared in the cross-section of calcined fly ash/EVA, indicating that the interaction force between calcined fly ash and EVA was much smaller than that of EVA itself, and the composites were more likely to break at the interface between calcined fly ash and EVA. A small amount of spherical particles appears on the section of ATO fly ash, which is due to the uniform coating of nano-level ATO on the surface of fly ash, resulting in a rough surface, so it has good compatibility with EVA. The surface of ATO fly ash/EVA has a more obvious drawing phenomenon than pure EVA, and the tensile strength is significantly enhanced in the mechanical property test. This phenomenon shows that the prepared ATO fly ash has good dispersion performance and improves the tensile strength of EVA. Therefore, the antistatic powder has a good application prospect in filling polymer materials.

## 4. Conclusions

(1) ATO was successfully coated on the surface of calcined fly ash, and the volume electrical resistivity of calcined fly ash was decreased from 1.72 × 10^12^ Ω·cm to 6 × 10^3^ Ω·cm, which belonged to the antistatic powder;
(2) According to the analysis of XRD, SEM, EDS and FTIR, Si-OH in fly ash and Sn (Sb)-OH of ATO are condensed to form a Si-O-Sn (Sb) bond, which is calcined at high temperatures to generate Sb-SnO_2_ with a rutile structure and conductive function. Nano-antimony-doped tin oxide particles are uniformly distributed on the surface of fly ash;
(3) The EVA-filling experiment showed that the tensile strength and elongation at the break of ATO fly ash/EVA were better than those of calcined fly ash/EVA. ATO fly-ash composite powder-filling EVA had little effect on the melting index. Compared with calcined fly ash, ATO fly ash has fewer spherical particles in the EVA section after filling, so it has better compatibility with EVA;
(4) The surface electrical resistivity of calcined fly ash filled with EVA is high, and its antistatic performance cannot meet the requirements. The surface electrical resistivity of EVA filled with ATO fly-ash composite powder is significantly reduced, which makes EVA have good antistatic properties. ATO fly ash can improve the antistatic performance of EVA without deteriorating the performance of the matrix;
(5) Compared with calcined fly ash, the limiting oxygen index of ATO fly ash composite powder filled with EVA increased more, which enhanced the flame retardant performance of EVA, indicating that there was a synergistic flame-retardant effect between fly ash and antimony-doped tin oxide.
(6) ATO fly-ash composite powder has a good application prospect in the field of polymer-material modification. The filled EVA can be widely used in the construction industry, wire and cable and other fields, which greatly reduces the harm caused by static electricity. ATO is expensive when used alone as an antistatic material, so coating it on economic materials such as fly ash can meet the antistatic requirements while reducing costs. The laboratory preparation method of this material is complicated, and its production process needs to be optimized during industrial production.

## Figures and Tables

**Table 1:** Chemical composition of fly ash (mass%).

Chemical Elements	Si	Al	Fe	Ca	K	Ti	Mg	Na	Sx	Else
Mass Fraction/%	50.00	23.67	9.34	6.25	4.00	2.38	1.19	0.723	0.685	0.017

**Table 2:** Performance analysis of EVA filled with composite power.

Sample	EVA	Calcined Fly Ash/EVA	ATO@fly Ash/EVA
Tensile strength (MPa)	6.10	6.38	6.50
Elongation at break (%)	434	323.6	409.23
Melt index (g/10 min)	2.90	5	2.50
Surface resistivity (Ω)	6.0 × 10^14^	1.95 × 10^14^	4.0 × 10^8^
Limit oxygen index (%)	19.8	22	23.5

## Data Availability

Data are contained within the article.
